# Guidelines to Support Nurse-Researchers Reflect on Role Conflict in Qualitative Interviewing

**DOI:** 10.2174/1874434600802010058

**Published:** 2008-06-10

**Authors:** Susan Jack

**Affiliations:** School of Nursing, McMaster University, Hamilton, Ontario, Canada

**Keywords:** Qualitative research, interviews, reflexivity, role conflict.

## Abstract

The conduct of a qualitative research interview is a complex social interaction that has the potential to influence, or be influenced by, both the researcher and the study participant. When a researcher is identified as a professional nurse, the identification of this role has the potential to influence the researcher-participant interaction. To understand the effect of a nurse-researcher’s involvement in an in-depth interview and on the data collected, issues to address include: clearly identifying the paradigmatic approach in which the research design is situated, examining the study participants' past experiences with research and the researcher’s profession, establishing appropriate boundaries with participants, deciding how to introduce the role of nurse-researcher to the participant and deciding if, or when, it would be appropriate to intervene within the research context. As nurse-researchers, professional knowledge and experiences have the potential to affect relationship development with study participants and obfuscate the purpose of the research interview. It is the researcher’s responsibility to participate in the activity of reflexivity to understand the effect of the nurse-researcher’s involvement on the data and make decisions that protect the participant’s integrity.

## INTRODUCTION

A nurse working in a clinical context who observes a mother struggling to latch her newborn to the breast, or watches an elderly patient having difficulty getting out of bed or hears an adolescent male express thoughts of suicide would not hesitate to appropriately intervene to support the client. But what if these events were precipitated by a study participant within the context of an interview being conducted by a nurse-researcher for the purpose of data collection in a study? What then would be the roles and responsibilities of the nurse-researcher and what would be the implications of providing nursing care during the process of data collection?

Conducting an interview in qualitative research is a complex social interaction that both the researcher and the respondent have the capacity to influence or be influenced by [1, 2]. The quality of the data that are shared and collected is influenced by multiple factors including the context of the interview, the meaning(s) attributed to being interviewed, and the values, beliefs, and experiences of both the researcher and the participant [2]. When a researcher is identified as a professional nurse, the identification of this role has the potential to influence the researcher-participant interaction [3, 4]. In this article, I will examine the relationship between nurse-researchers and research participants within the context of qualitative interviews. Issues that arise during interviews related to role conflict and the desire to provide clinical interventions are discussed. Questions for consideration are proposed to assist novice qualitative nurse-researchers reflect about the nature of the relationship they establish during an interview so that both the credibility of the data and the participant's integrity can be maintained.

## THE DILEMMA FOR NURSE- RESEARCHERS

In quantitative research, interviews are conducted to complete structured questionnaires. Here the role of the researcher is clear: to remain neutral and objective and to limit researcher influence over the study subject. In contrast to survey research, qualitative interviews are conversational in nature and the goal is to discover how the phenomenon under study is perceived and described by the participant in his or her own words. However, because the qualitative interviewer is the research instrument through which data are collected, filtered and processed, it is his/her values and beliefs that influence what concepts should be further explored [5, 6]. Despite the reliance on in-depth interviewing in qualitative research, few nurse-researchers include a detailed discussion in published works about the nature of the relationship with research participants or particular challenges encountered during interviewing [2].

There are anecdotal examples though of qualitative researchers who describe the struggles they experienced in balancing their dual roles as both researcher and clinician. Two physicians, Hamberg and Johansson [7] discuss the tension, confusion, and power asymmetry that results when female physicians interview their own female patients in an attempt to understand the life experiences of women with painful, undefined musculoskeletal disorders. Sword [8] in her interviews with low-income women about their perceptions and use of prenatal health care services acknowledges the difficulty in separating the researcher role from the nurse role and the ethical dilemmas encountered by wanting to provide health information to address the participants’ questions. In my own interviews with high-risk mothers about the process of engaging with public health nurses during home visits frequently the focus of the interaction would move from researcher directed data collection to the participants emotionally sharing stories of their chronic and multiple stresses experienced daily [9]. In each situation, the researchers were left reflecting about the appropriateness of intervening and the impact of such actions on data collection and analysis.

## PHILOSOPHICAL ASSUMPTIONS

It is generally accepted that effective qualitative researchers require strong interviewing skills including the ability to quickly establish rapport during the initial contact [10]. Good rapport is essential to building trust, which is necessary if participants are to share intimate details about private, and often sensitive or controversial, aspects of their lives [11]. Where trust does not exist, participants are more likely to provide information based on what they perceive the researcher wants to learn [5].

Some authors state that while this trust is necessary to enhance the depth of the information collected, it must be balanced with a certain level of detachment so that partial objectivity can be achieved [1,12]. This requires that the researcher refrain from offering opinions or information during the course of the interview, so as to not introduce bias. Others assert that meaningful data can only be obtained when the researcher and participant establish an authentic relationship built on closeness, engagement, reciprocity and mutual self-disclosure [4, 13, 14].

This debate is important because at the foundation of qualitative research there is a continuum of philosophical beliefs, each based on different assumptions. Prior to commencing qualitative interviewing, it is important that nurse-researchers are familiar with the philosophical assumptions underlying qualitative research. Different epistemological assumptions exist about the nature of the researcher-participant relationship in qualitative interviewing. While there is general acknowledgement that qualitative research cannot be purely objective because the research questions, design, and analysis are all influenced by the researcher's experiences, strategies to minimize the influence of the researcher on the interaction have been emphasized [1, 10]. Debate regarding the dominance of this approach has resulted in the “relative neglect of the impact of the person of the researcher on data gathering and analysis” [5].

Proponents of interpretive and critical paradigms reject the possibility that value-free, objective and neutral researcher-participant relationships can be developed within the context of a qualitative interview. They suggest that an openly subjective approach be adopted in qualitative research wherein the researcher participates in the interview as a whole person and strives to develop authenticity through forming a relationship with the participant built on openness, respect and reciprocity [4, 14]. It is thus openly acknowledged that the interviewer is part of the research process and that his/her values and beliefs will influence the information that is shared and collected. This approach has been strongly advocated by feminist researchers who believe that if meaningful information is to be shared, then personal involvement “is the condition under which people come to know each other and to admit others into their lives” [15].

## ISSUES IN QUALITATIVE INTERVIEWING 

### Role Conflict

The role of a qualitative researcher is to collect, analyze and interpret data, and report findings for the purpose of increasing understanding about the phenomenon under study [1]. However, within the context of an interview, it may become difficult to maintain that primary identity or role of researcher. This problem has been well documented in the nursing literature on qualitative research (e.g. [16, 17]). In a study of family caregiving conducted by nurse-researchers, multiple roles and relationships between the researchers and the participants emerged, including: stranger-stranger, researcher-participant, friend-friend, nurse-client, and guest-host [13].

Nurse-researchers may experience role conflict when deciding how to introduce themselves to participants. Interviewers are aware that the initial introduction will influence the participant's perception of them [14, 16, 18]. For example, if they identify themselves as 'nurse-researchers', then participants with positive past experiences with nurses may feel comfortable sharing intimate information to which others may not have been privy [19]. Conversely, if participants' experiences have been negative then they may purposefully omit information, especially if they are aware that the nurse has a professional responsibility to act on any disclosed information. If participants are unfamiliar with the process of research, then they may view the interviewer in the more familiar role of 'nurse' and attempt to focus the interaction towards their clinical concerns [3, 20]. In a study to explore the influence of professional role on qualitative interviews, Richards and Emslie [21] compared researcher-participant interactions in two different qualitative studies about middle-aged adults’ perceptions of heart disease. One study was conducted by a sociologist who introduced herself as a researcher with no medical background; the second study was conducted by a General Practitioner (GP), who identified herself as a non-practicing physician. In the study conducted by the sociologist, participants expressed views that were more critical of physicians, whereas in the interviews conducted by the GP, some participants were apologetic about expressing negative views about physicians and several took the opportunity in the interview to ask the physician medically related questions. Richards and Emslie [21] conclude that disclosure of the researcher’s professional role and the participant’s perceptions of that role have the capacity to influence the qualitative data shared. On the other hand, concealing one's role as a clinician may create both personal and ethical concerns for some researchers [5, 8].

An extra layer of complexity exists if the researcher conducts research interviews with his/her own patients. Hamberg and Johansson [7], family physicians who interviewed their patients about long-term musculoskeletal pain, found that interview situations were filled with tension and that power asymmetry with participants was extreme. Both Britten [22] and Archbold [20] advise that clinicians should not interview their own patients because they may feel compelled to participate, fearing that refusal might jeopardize the care or treatment they are receiving.

### The Desire to Provide Clinical Interventions

The ability to effectively communicate and interpret data on multiple levels is a skill that contributes to both effective in-depth interviewing and the delivery of professional health care [5]. However, even though the goal of a research interview is to generate knowledge, the act of participating in this process may have therapeutic benefits for the interviewee. For some people, especially those who are vulnerable and marginalized, the experience of telling their story has been described as empowering, cathartic, healing and an opportunity for self-reflection [23, 24].

A different situation exists when a nurse-researcher decides to intervene or is asked to provide health information during an interview. In a study about the transition to motherhood, Oakley [15] identified that over the course of 178 interviews, she was asked 878 questions and that 76% of these questions were requests for health information. Several authors argue that while health care providers are socialized to care and provide service, the primary objective of a research interview is to collect data and not to offer intervention [10, 19].

The impact of providing clinical interventions during in-depth interviewing can be interpreted differently depending upon the researcher's philosophical assumptions about the nature of the researcher-participant relationship. From a post-positivist perspective, Hutchinson and Wilson [25] caution that interventions made during an interview threaten the validity or objectivity of the data. Information provided by the nurse-researcher may influence the participants’ responses, change the focus of the interview, discourage the participant from openly sharing more information or prematurely terminate the interview [5, 10]. In spite of this, refusing to answer clinical questions or concerns may negatively affect the interview [22]. One strategy to deal with these issues is to defer requests for information until the end of the interview [22]. When a need for further intervention is identified, then the nurse-researcher should refer the participant to another health care professional [10].

In interpretative or critical paradigms the subjective nature of the researcher-participant relationship is highlighted. Therefore, intervening during an interview is seen as enhancing rather than threatening the validity of the data [15, 26]. Rather, it is this reciprocal exchange of information that builds rapport and trust so that more meaningful information is ultimately shared [11, 27]. Offering of health-related information is also seen as a positive reward that offsets the burden of participating in long interviews that are often physically and emotionally exhausting [15, 28]. Wilde [4] hypothesizes that intervening may even open up new areas for inquiry and exploration. Additionally, qualitative researchers who apply a feminist methodological lens to the research process identify that the research process can be utilized as a vehicle to empower change at individual and system levels [11]. In a feminist grounded theory study to explore family health promotion practices by mothers with a history of intimate partner violence, Ford-Gilboe, Wuest and Merritt-Gray [29] identified that in addition to providing information on community resources at the end of the interview, they also empowered the women to access those resources and in some cases advocated directly on the behalf of the study participant.

Regardless of the researcher's paradigmatic stance, there are certain circumstances when the clinician has a legal and ethical responsibility to intervene and perhaps even to stop an interview. If a serious conflict should develop, then the need to provide a therapeutic intervention should take precedence over the need to collect data [30]. For example, if during the course of an interview, the researcher learns or suspects that a child may be in need of protection, the interviewer must report this information to a local child welfare agency. The nurse-researcher also has a professional responsibility to intervene if there is an immediate threat to an individual's health and safety, such as respiratory distress or cardiac arrest and she/he has the professional skills to cope with such a threat. The nurse-researcher may also feel a professional responsibility to intervene if she is interviewing an individual in the community who has limited access to health and social services, or who may not have the skills to follow through with a referral to another health care professional.

To facilitate the implementation of ethical research practices in qualitative research, Hewitt [18] has developed a comprehensive list of the components of ethical researcher-participant relationships. This framework provides researchers with a description of the factors of the research relationship that should be addressed in order to ethically acknowledge bias, maintain rigor, establish rapport maintain respect for participant autonomy and confidentiality and how to avoid exploitation.

## QUESTIONS FOR PERSONAL REFLECTION TO AID DECISION-MAKING

No absolute answers can be given to resolve many of the challenges that nurse-researchers experience while conducting qualitative interviews. Instead, researchers need to reflect upon, rather than ignore, the effect of their involvement on the data [31]. Reflexivity, or reflexive analysis, is the process whereby the researcher evaluates the self as the data collection instrument and analyzes the influence of personal and professional values, beliefs and experiences that impinge on the research [5, 28]. Arber [16] asserts that the use of reflexivity in qualitative research facilitates the process of examining the impact of the nurse-researcher on all aspects of the study process and the collected data.

There is a considerable amount of literature describing and highlighting the value of reflexivity. Finlay [32] provides a detailed description of how reflexivity can be applied across the entire qualitative research process, but there is little to guide nurses who struggle with balancing a dual role while conducting qualitative interviews. What follows is a list of questions and suggestions to assist nurse-researchers anticipate, reflect on, and resolve some of the issues that arise around role conflict and the dilemma of intervening within the context of an in-depth interview.

### 1. What is the Paradigmatic Approach in which the Research Design is Situated?

In qualitative research it is imperative that the researcher explicitly identifies the paradigmatic approach in which the research design is situated. The assumptions associated with a particular paradigm will define how a researcher interacts with participants. Related questions that need to be raised include: What is the form and nature of reality? What is the nature of the relationship between the nurse-researcher and the participant?

### 2. What have been Participants' Past Experiences with Research and the Researcher’s Profession?

It is important to explore with the participant their perceptions and experiences with past care from health and social service providers. The participant's perception of the agency sponsoring the research should also be examined. The nature and quality of their previous experiences with staff from that agency also needs to be understood. Particularly in program evaluation studies, if the nurse-researcher is a part of the institution where the participant is receiving care then honest opinions may be suppressed [33].

### 3. What Kind of Boundaries should be Established Between the Nurse-Researcher and the Participant?

It is impossible to reduce all inequalities of power, but researchers must protect participants' rights to anonymity, confidentiality and reduce any psychological, physical or social risks associated with participating in an interview [24]. To decrease the imbalance of power, some authors advocate for mutual self-disclosure, or the sharing of personal values, beliefs or opinions to emphasize shared experiences and minimize differences [4, 15]. When the researcher expresses opinions there is a risk that the participant will choose to agree with the researcher's conclusions, but Hamberg and Johansson [7] suggest that sharing opinions and interpretations actually opens up opportunities for the participants to react, protest or modify their responses. If the researcher chooses to disclose personal information, how much is appropriate? Self-disclosure and the intimate content of many interviews may turn the researcher-participant relationship into a friend-friend relationship [3, 15]. Does this enhance the quality of the data or does it create an increased risk of exploitation [11]? How does the development of a friendship affect the reporting of research findings?

### 4. How should a Nurse-Researcher Present his/her Role to a Participant?

To minimize role conflict, researchers need to be able to clearly define and articulate their roles to participants. Careful consideration must be given to how one presents him or herself to the participant: is it as a graduate student, researcher or nurse-researcher? The participant's beliefs about the role will influence what information is disclosed [14]. For example, if identified as a nurse-researcher, what is the participant's perception of a researcher and of a nurse? Different factors may influence an individual's decision to interact with a nurse versus a researcher. If the participant perceives the nurse-researcher in the more familiar role of 'nurse', May [34] argues that this is an obstacle in obtaining informed consent. However, it has been discussed that it is often a challenge in interactions between nurse-researchers and study participants to suppress acknowledgment of the ‘nurse’ identity [35, 36]. Colbourne and Sque [35] conclude therefore that the researcher’s identity as a nurse should be revealed but that the impact of this revelation on the data be reflected upon.

### 5. Should a Nurse-Researcher Deliver Health Care Interventions During an Interview?

The decision to intervene will be determined by the underlying assumptions of the research design and by who identifies the need for intervention and the immediacy of the need. If a life-threatening situation arises, then the nurse- researcher must intervene immediately; other interventions could probably be left until the end of the interview. Sponsoring agencies should be aware that clinician-researchers might provide clinical interventions during an interview. If this is the situation, it is the researcher’s responsibility to be aware of the agency’s policies and procedures, have a method of reporting interventions if necessary and ensure that they have adequate insurance coverage for their professional interventions.

If the decision has been made that interventions will not be offered within the interview, then the researcher should be knowledgeable of community resources to which to refer the participant or be able to leave a package of pertinent information with the participant. If there is the potential that the content of the interview will trigger a negative emotional response from the participant, then the researcher has an ethical duty to have a counselor or other services available for follow-up. It would be important to identify if the sponsoring agency has the resources available to respond to an increase in workload in such situations.

### 6. What Impact did the Intervention have on the Nature of the Relationship?

Any decisions to intervene should be documented in the researcher's field notes and described in research publications [27]. Additional questions for reflection could include: Did the intervention open up alternate areas for inquiry [4]? What effect will intervening have on the researcher's relationship with other clinicians or the participant's relationship with his/her primary health care provider? Does the researcher have an obligation to follow-up and evaluate the effectiveness of the intervention?

## CONCLUSION

One of the hallmarks of qualitative interviews is that the interviewer is the research instrument through which data are filtered and processed. This means that it is not only inadvisable but also impossible to have a value-free, impersonal researcher-participant interaction during a research interview. There is a need for continued open and honest discussion about the realities of conducting qualitative interviews by clinician-researchers. Anecdotes in the literature reveal that experienced researchers do struggle with balancing data collection with their role of health care provider and that some do clinically intervene within the context of an interview. Nurses, as part of their educational preparation, develop skills in communication, teaching and counseling and therefore find themselves providing health care to study participants, particularly if the intervention involves health education. In addition, when these skills are appropriately incorporated into a qualitative interview the result is the development of a more meaningful relationship with the participant.
